# Ionizing radiation regulates cardiac Ca handling via increased ROS and activated CaMKII

**DOI:** 10.1007/s00395-013-0385-6

**Published:** 2013-09-26

**Authors:** Can M. Sag, Hendrik A. Wolff, Kay Neumann, Marie-Kristin Opiela, Juqian Zhang, Felicia Steuer, Thomas Sowa, Shamindra Gupta, Markus Schirmer, Mark Hünlich, Margret Rave-Fränk, Clemens F. Hess, Mark E. Anderson, Ajay M. Shah, Hans Christiansen, Lars S. Maier

**Affiliations:** 1 Department of Cardiology and Pneumology/Heart Center, Georg-August-University Göttingen, Robert-Koch-Str. 40, 37075 Göttingen, Germany; 2 British Heart Foundation Centre of Excellence, King’s College London, James Black Centre, London, UK; 3 Department of Radiotherapy and Radiooncology, Georg-August-University Göttingen, Göttingen, Germany; 4 Department of Clinical Pharmacology, Georg-August-University Göttingen, Göttingen, Germany; 5 Cardiovascular Research Center, Carver College of Medicine, University of Iowa, Iowa City, USA; 6 Department of Radiotherapy and Oncology, Hannover Medical School, Hannover, Germany

**Keywords:** Irradiation, Excitation–contraction coupling, Calcium, Free radicals, CaMKII

## Abstract

Ionizing radiation (IR) is an integral part of modern multimodal anti-cancer therapies. IR involves the formation of reactive oxygen species (ROS) in targeted tissues. This is associated with subsequent cardiac dysfunction when applied during chest radiotherapy. We hypothesized that IR (i.e., ROS)-dependently impaired cardiac myocytes’ Ca handling might contribute to IR-dependent cardiocellular dysfunction. Isolated ventricular mouse myocytes and the mediastinal area of anaesthetized mice (that included the heart) were exposed to graded doses of irradiation (sham 4 and 20 Gy) and investigated acutely (after ~1 h) as well as chronically (after ~1 week). IR induced a dose-dependent effect on myocytes’ systolic function with acutely increased, but chronically decreased Ca transient amplitudes, which was associated with an acutely unaltered but chronically decreased sarcoplasmic reticulum (SR) Ca load. Likewise, in vivo echocardiography of anaesthetized mice revealed acutely enhanced left ventricular contractility (strain analysis) that declined after 1 week. Irradiated myocytes showed persistently increased diastolic SR Ca leakage, which was acutely compensated by an increase in SR Ca reuptake. This was reversed in the chronic setting in the face of slowed relaxation kinetics. As underlying cause, acutely increased ROS levels were identified to activate Ca/calmodulin-dependent protein kinase II (CaMKII). Accordingly, CaMKII-, but not PKA-dependent phosphorylation sites of the SR Ca release channels (RyR2, at Ser-2814) and phospholamban (at Thr-17) were found to be hyperphosphorylated following IR. Conversely, ROS-scavenging as well as CaMKII-inhibition significantly attenuated CaMKII-activation, disturbed Ca handling, and subsequent cellular dysfunction upon irradiation. Targeted cardiac irradiation induces a biphasic effect on cardiac myocytes Ca handling that is associated with chronic cardiocellular dysfunction. This appears to be mediated by increased oxidative stress and persistently activated CaMKII. Our findings suggest impaired cardiac myocytes Ca handling as a so far unknown mediator of IR-dependent cardiac damage that might be of relevance for radiation-induced cardiac dysfunction.

**Electronic supplementary material:**

The online version of this article (doi:10.1007/s00395-013-0385-6) contains supplementary material, which is available to authorized users.

## Introduction

Chest radiotherapy is an integral part of prevalent multimodal therapy concepts for malignant diseases, e.g., breast, esophageal, lung cancer, or thoracic lymphoma [9]. The main aim of modern high-precision radiation therapy is to kill cancer cells without damaging surrounding healthy tissue, a clinical approach that involves the precisely targeted delivery of ionizing radiation [and the subsequent formation of intracellular reaction oxygen species (ROS)] to targeted tissue. However, even intensity modulated radiotherapy cannot avoid a certain radiation exposure of normal tissue nearby.

In this context, growing evidence suggests that thoracic radiotherapy may cause clinically relevant cardiac toxicity. While initially radiation-induced pericardial disease and the effect of irradiation on endothelial cells leading to coronary artery diseases were in the center of analysis [29], it is now accepted that irradiation can also lead to cardiomyopathy, valvular heart disease, and conduction abnormalities [1]. First, clinical data were gained from long-term survivors of Hodgkin’s lymphoma treated with chest radiotherapy, in which a significantly increased risk of death due to heart diseases was found [11]. Similar data were reported for patients undergoing radiotherapy for lung cancer [7]. Nowadays, special attention is also paid to females treated with adjuvant radiotherapy for left-sided breast cancer as it has been shown that these patients also have an increased risk of chronic cardiac morbidity [17] due to a significant radiation exposure to the heart [4]. While the threshold dose for irradiation-induced cardiac morbidity remains controversial, tolerance doses recommended today are 40 Gy and are thus similar to other radiosensitive organs [25]. Recent studies suggest that cardiac damage may already arise from radiation doses of 15 Gy, which is much less than previously assumed [18]. In contrast to the clinical relevance and the need for development of therapeutic strategies, the underlying pathomechanisms of radiation-induced cardiac dysfunction are still poorly understood.

Radiation-induced cardiomyopathy (RICM) is clinically characterized by a rather mildly decreased left ventricular ejection fraction, but severely impaired diastolic function [6, 13] that usually arises years after treatment. Previous studies have mainly focused on vascular injury as the main mechanism responsible for radiation-induced cardiac damage [12]. In that concept, cardiocellular dysfunction is suggested to be an indirect effect secondary to vascular injury. However, little is known about potentially direct effects of irradiation on the myocardium and on its core units, single cardiac myocytes.

Physiological cardiac function with regular systolic contraction and diastolic relaxation of the heart largely depends on intact Ca handling of cardiac myocytes summarized as excitation–contraction coupling [3]. In turn, disturbed Ca handling is a hallmark of cardiac dysfunction [19]. Growing evidence suggests that increased cardiocellular ROS (or oxidative stress) can induce severe contractile dysfunction in cardiac myocytes by impairing cardiac Na and Ca handling [24]. Since irradiation is known to induce the formation of ROS in target tissue and can lead to subsequent cardiac dysfunction, we speculated whether ROS-dependently impaired cardiac myocytes Ca handling might be a direct consequence of ionizing radiation independent of effects secondary to vascular damage [12] that would represent an additional pathomechanisms by which IR might affect cardiocellular function.

## Materials and methods

### Irradiation procedure and animal husbandry

Single doses of radiation (sham i.e., 0, 4 and 20 Gy) were targeted to the mediastinal area of anesthetized black swiss wild-type mice (2 % isoflurane vaporized in 100 % oxygen using positive pressure ventilation) by shielding the rest of the animal’s body with lead plates. To assess ‘late’ effects of IR on cardiac function, some mice were held in individually ventilated cages with unrestricted access to food and water for another week following irradiation. Isolated cardiac myocytes (see below) were irradiated after plating them onto superfusion chambers. Irradiation was executed at room temperature with single doses of 4 and 20 Gy, respectively. An X-ray generator (Gulmay RS225 GS014, Gulmay Medical Ltd, Camberley, Surrey, Great Britain) with a 0.5-mm Cu filter was used operating at 200 kV constant potential and 15 mA with a dose-rate of 1 Gy/min. “Acute” measurements were performed from ~20 min up to ~2 h after IR, while “Chronic” measurements were performed 1 week after IR. Sham-irradiated mice and cells served as specific controls in all experiments.

### Myocyte isolation and culture

Cardiac myocytes were isolated from mice that were anesthetized in an inhalation chamber with isoflurane at a dose of ~20 μl/g bodyweight before hearts were excised [22]. Explanted hearts were retrogradely perfused with a Ca-free Tyrodes’ solution containing (in mM) NaCl 113, KCl 4.7, KH_2_PO_4_ 0.6, Na_2_HPO_4_·2H_2_O 0.6, MgSO_4_·7H_2_O 1.2, NaHCO_3_ 12, KHCO_3_ 10, HEPES 10, taurine 30, BDM 10, glucose 5.5, phenol-red 0.032 (37 °C, pH 7.4). 7.5 mg/ml liberase 1 (Roche diagnostics, Mannheim, Germany), trypsin 0.6 % and 0.125 mM CaCl_2_ were added to the perfusion solution. Perfusion was continued until the heart became flaccid. Ventricular tissue was removed, cut into small pieces and dispersed. Ca reintroduction was performed by step-wisely increasing [Ca] from 0.1 to 0.8 mM. Cells were plated onto superfusion chambers, with the glass bottoms treated with laminin. For some experiments, myocytes were further cultured. Cells were initially plated for 2 h in minimal essential medium (Sigma-Aldrich, Germany) including (in mM) l-glutamine 2, BDM 10, penicillin 200 U/ml, streptomycin 0.2 mg/ml, linoleic/oleic acid 0.1 μL/mL, and BCS 5 %. Afterwards, medium was exchanged for culture medium containing the same ingredients as plating medium but without BCS. Culture was performed for 24 ± 4 h (37 °C, 5 % CO2) [21]. All investigations conform to the “Guide for the Care and Use of Laboratory Animals” published by the US NIH (Publication No. 85-23, revised 1996) and were approved by the Ethics Committee of the Medical Faculty of the University of Goettingen.

### Epifluorescence microscopy

We used a Nikon Eclipse TE2000-U inverted microscope provided with an IONOPTIX fluorescence detection system for assessment of [ROS]_i_, simultaneous [Ca]_i_ and shortening measurements, as well as [Na]_i_-assessment as previously described. ROS-detection was performed at 535 ± 20 nm using 10 μmol/L CM-H_2_DCFDA [22]. Please note that ROS-measurements as shown in Fig. 6a, b were obtained in the presence of a ‘1.0 grey filter’. For Ca-measurements, Fura-2 AM was used whereas SBFI served as an indicator of intracellular Na-concentrations [31] (both 10 μmol/L, with alternate excitation at 340 and 380 nm). Ca transient amplitude was assessed as the 340/380 nm fluorescence ratio [F340/F380 nm in ratio units (ru)]. Myocytes were field-stimulated (~20 V) at 1 Hz for 5–10 min until steady-state conditions were achieved. Myocytes were superfused with a normal Tyrode’s (NT) solution containing (in mM) NaCl 140, KCl 4, glucose 10, HEPES 5, MgCl_2_ 1, CaCl_2_ 1 at 37 °C and pH 7.4 [22]. Cells were transilluminated by red light (>650 nm) to visualize myocytes sarcomeres, and shortening was measured using a sarcomere length detection system to asses the ratio between systolic and diastolic (or resting) cell length. Based on these measured lengths, we calculated fractional shortening (in % of resting cell length, %RCL). SR Ca load was evaluated by Ca transient amplitudes induced by rapid application of caffeine (10 mmol/L). In some experiments, the reverse mode of Na/Ca exchanger (NCX) was blocked by 5 μmol/L KB-R7943 [31].

### Confocal microscopy

To asses spontaneous Ca release events from the SR (Ca sparks) myocytes were loaded with Fluo-4 (10 μM) and mounted on a laser scanning confocal microscope (Zeiss LSM 5 PASCAL, Göttingen, Germany) [22]. Fluo-4 AM loaded cells were excited via an argon laser (at 488 nm) and emitted fluorescence was collected at 505 nm through a long-pass emission filter. Cells were superfused with NT solution. Stimulation frequency was 0.5 Hz. Ca sparks were analyzed by the use of Zeiss software.

### Echocardiography

*In vivo* cardiac function in live animals was assessed acutely and chronically using a high-frequency echocardiography system specifically designed for small animal studies (Vevo2100, Visualsonics, Canada) [28]. Echocardiography was performed under anesthesia induced and maintained at different doses of isoflurane (5 and 1 % for fast induction and maintenance, respectively) vaporized in 100 % oxygen delivered at 1.5–2 L/min. Heart rate was kept at ~400–450 beats per minute while respiratory rate was ~100 breaths per minute. Body temperature was ~36.5 ± 1 °C throughout the examination. Scanning was performed using an echo transducer of 30 MHz in frequency. Two-dimensional images in parasternal long-axis view were obtained and saved as video loops that consisted of 300 frames for the estimation of LV systolic function, contractility and dimensions. Systolic function was measured using the semi-automatic 2D tracing method which measures the end-systolic and end-diastolic volume of the LV as well as the ejection fraction as parameters of global systolic function. Contractility of the LV walls was characterized using the VevoStrain modality incorporated into the Vevo system, which generates strain parameters in longitudinal as well as radial dimensions from the semi-automatic tracing of the endocardium of the LV [2].

### Electron spin resonance spectroscopy

Electron spin resonance (ESR) spectroscopy was performed to measure ROS generation in cardiac myocytes following irradiation. Measurements were carried out using an e-scan machine (Bruker, Karlsruhe, Germany) with 1-hydroxy-3-methoxycarbonyl-2,2,5,5-tetramethylpyrrolidine (CMH, Noxygen, Elzach, Germany) as spin probe which has particular sensitivity to detect global ROS [8]. Cardiomyocytes were washed and resuspended in ESR buffer (Noxygen), supplemented with CMH at 200 μmol/L and divided into three portions (0, 4 and 20 Gy) immediately prior to irradiation. ROS levels were normalized to cell volume (in pL) based on standard volumes for adult cardiac mouse myocytes [3]. ESR settings were as follows: magnetic field center 3,386 G, sweep 9 G, microwave frequency 9.51 GHz, microwave power 21.9 mW, modulation frequency 86 kHz, modulation amplitude 2.6 G, modulation phase 359°, time constant 41 ms, conversion time 10.2 ms, and sweep time 5.2 s.

### ROS-scavenging and CaMKII-inhibition

CaMKII-inhibition was ensured before irradiation was applied by incubating isolated cardiac myocytes with the organic CaMKII-inhibitor KN-93 (1 μmol/L) or myristoylated autocamtide-2 related inhibitory peptide (AIP, 5 μmol/L) [23]. Since KN-93 has well known unspecific side effects we used the inactive analog KN-92 (1 μmol/L, without CaMKII-inhibiting potential) for comparison [22]. For ROS-scavenging experiments, isolated myocytes were pre-treated for 15 min with 1 mmol/L of the scavenger drug melatonin (Sigma-Aldrich, Germany). Afterwards, cells were exposed to irradiation and measured as described above.

### Immunoblotting

We performed standard immunoblotting to investigate the expression and phosphorylation levels of Ca handling proteins in homogenized cardiac tissue following irradiation. For detection of CaMKII protein expression, we used antibodies kindly provided by Dr. Bers, (UCD, Davis, CA, USA). CaMKII-phosphorylation levels were detected using a phosphospecific CaMKII-antibody (1:1,000, Thermo scientific). Oxidized CaMKII was detected using an immune serum against oxidized M281/M282 of CaMKII (ox-CaMKII) [10, 22]. PLB-expression (1:10,000, Millipore) and RyR2-expression (1:10,000, Sigma-Aldrich) were measured along with their individual phosphorylation status at Thr-17 (1:10,000, Badrilla), at Ser-16 (1:10,000, Badrilla, for PLB), at Ser-2809 (1:5,000, Badrilla), and at Ser-2814 (1:5,000, Badrilla, for RyR2). SERCA2a (1:20,000, AffinityBioReagents) and NCX protein expression (Swant 1:5,000) were measured as well. Secondary antibodies were purchased from GE Healthcare. Each expression or phosphorylation signal was normalized to its individual loading control (GAPDH). The phosphorylation status of a protein is, therefore, expressed as ‘(protein-phosphorylation/GAPDH)/(protein-expression/GAPDH)’. Values were afterwards normalized to ‘0 Gy control’.

### Statistics

**Fig. 1:**
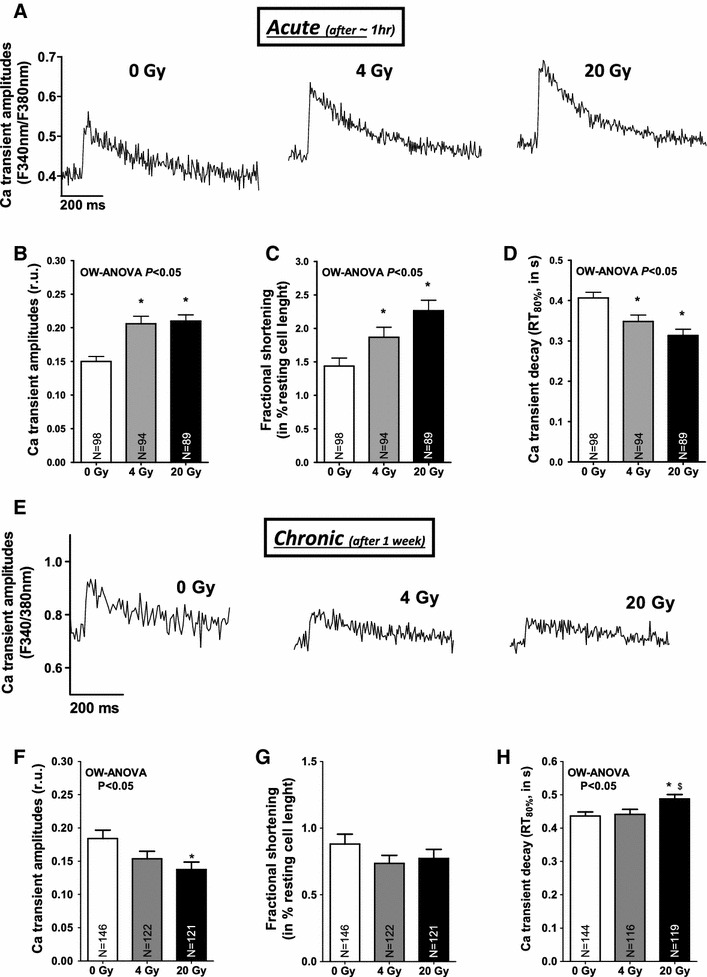
Irradiation biphasically regulates cardiac Ca handling. **a** Original Ca traces obtained from three different cardiac myocytes showing acutely increased Ca transient amplitudes following irradiation. Average data for **b** Ca transients, **c** fractional shortening, and **d** Ca transient decay. **e** Depicts original Ca traces from myocytes in the chronic setting. Mean data for **f** Ca transients, **g** fractional shortening and **h** Ca transient decay. One-way analysis of variance (OW-ANOVA) indicates dose-dependent effects of irradiation. *Significance vs. 0 Gy and ^$^significance vs. minor irradiation dose using Newman–Keuls post hoc test

**Fig. 2:**
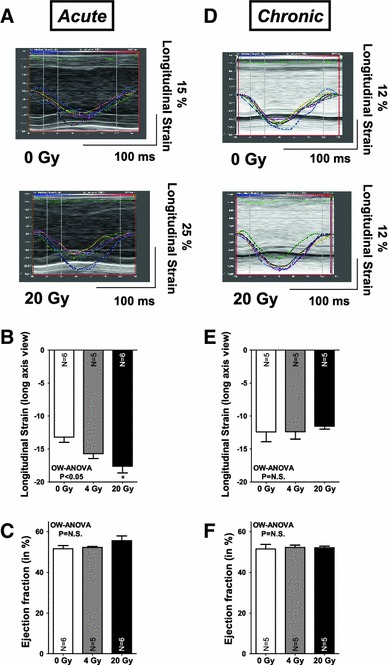
Biphasical regulation of LV contractility following irradiation. Representative measures of longitudinal strain as shown in **a** (acutely) and **d** (chronically) demonstrate acutely increased LV contractility to decline in the long-term. Mean values for longitudinal strain are shown in **b, e** and in **c, f** for ejection fraction. Echocardiographic findings are summarized in Tables 1 (acute) and 2 (chronic setting)

**Fig. 3:**
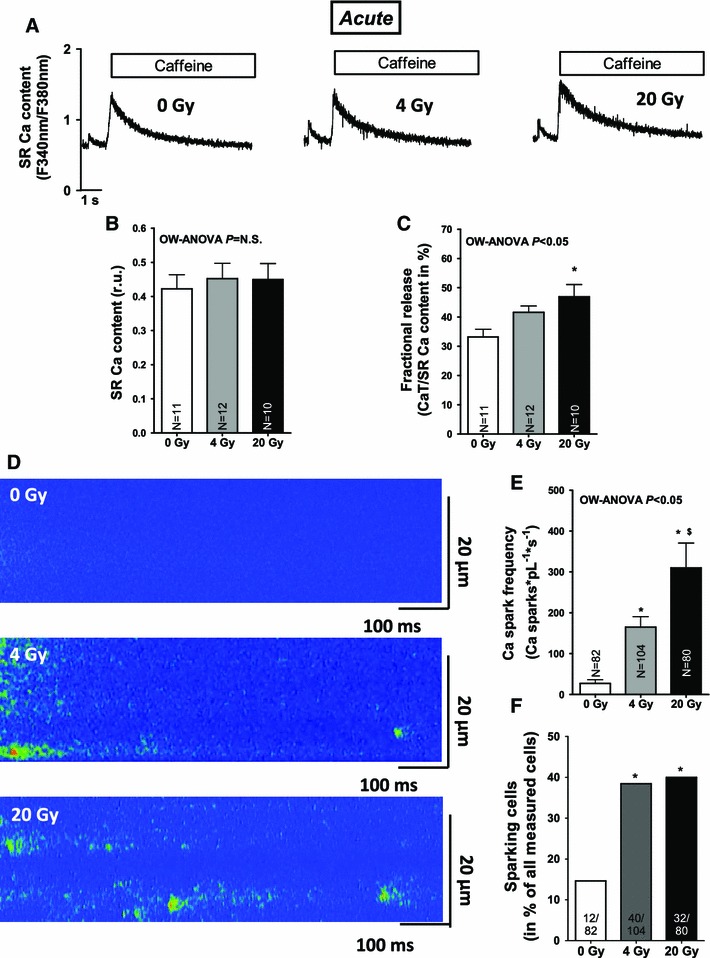
Irradiation acutely enhances SR Ca release in cardiac myocytes. **a** Representative Caffeine-induced Ca transients showing acutely unaltered SR Ca content. Average values for **b** SR Ca content and **c** fractional release. **d** Shows an increased Ca spark frequency on confocal line-scan images following irradiation. Average values for **e** Ca spark frequency, **f** percentage of sparking cells

**Fig. 4:**
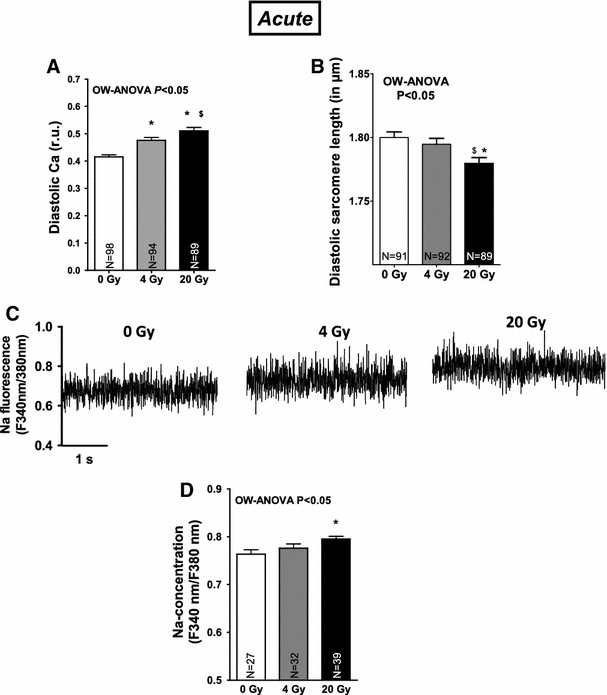
Na-dependent Ca overload and decreased sarcomere lengths following irradiation. Average values for **a** diastolic Ca and **b** diastolic sarcomere lengths. **c** Shows increased Na fluorescence following irradiation. Mean values are presented in **d**

**Fig. 5:**
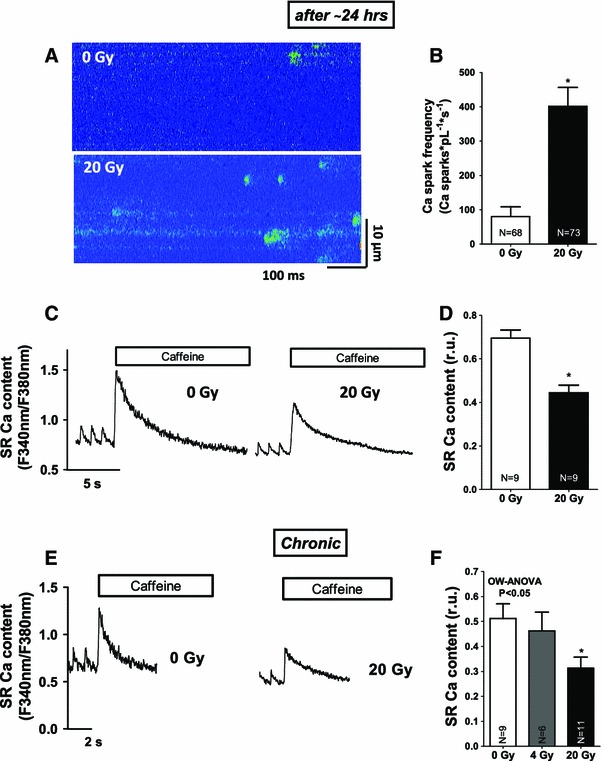
Persistent SR Ca leakage and subsequent SR Ca depletion in the long-term following IR. **a** Original confocal line-scan images as obtained from cultured myocytes show persistently increased SR Ca loss following irradiation (after 24 h). **c** Shows cultured, irradiated cells with decreased SR Ca content. Mean values for **b** Ca spark frequency and **d** SR Ca content. **e** Shows original Caffeine-induced Ca transients reflecting decreased SR Ca content in the chronic setting. Average values are shown in **f**

**Fig. 6:**
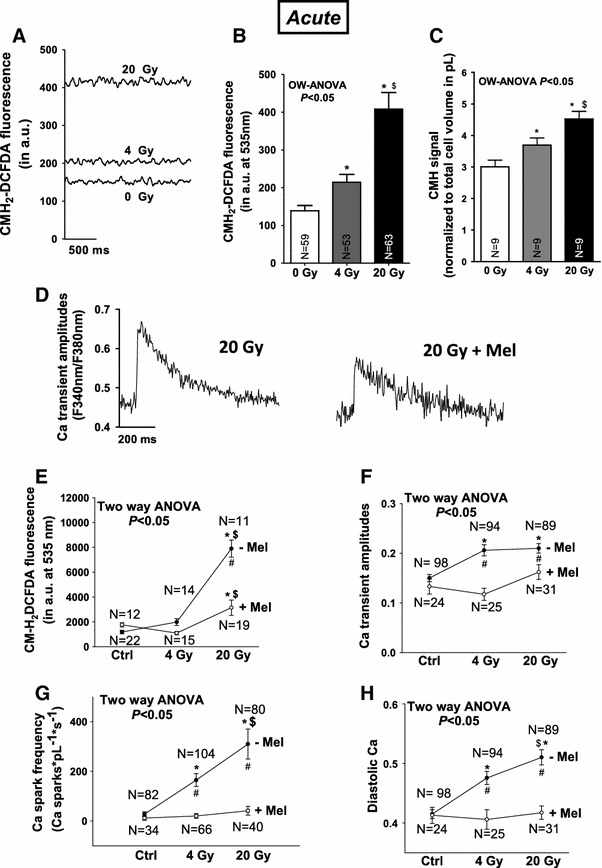
Irradiation increases oxidative stress to regulate cardiac Ca handling. **a** Original CM-H_2_DCFDA fluorescence shows dose-dependently increased ROS following irradiation. **b** Average data for CM-H_2_DCFDA fluorescence and **c** ESR-measurements. **d** Original Ca transient traces with or without Melatonin pretreatment demonstrating attenuated Ca transient amplitudes in case of ROS-scavenging. **e** Decreased CM-H_2_DCFDA-fluorescence in case of ROS-scavenging using Mel. Average values for **f** Ca transient amplitudes, **g** Ca spark frequency, and **h** diastolic Ca. ^#^Indicates significance vs. corresponding dose in case of melatonin pretreatment using Newman–Keuls post hoc tests while two-way analysis of variance (TW-ANOVA) indicates significance vs. melatonin pretreated cells

**Fig. 7:**
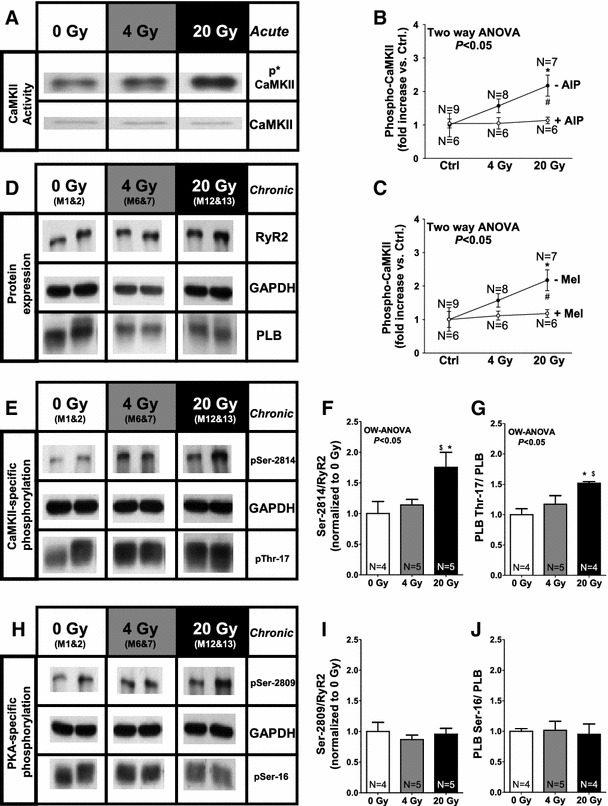
Irradiation activates CaMKII**. a** Original Western Blot images show activated CaMKII as measured by phosphospecific CaMKII-antibodies. **b** Mean data for phospho-CaMKII with and without CaMKII-inhibition (AIP) and **c** for phospho-CaMKII demonstrating prevention of CaMKII-activation in case of ROS-scavenging. **d** Shows original protein expression for RyR2 and PLB. **e** Shows CaMKII-dependent and **h** PKA-dependent phosphorylation of the RyR2 and PLB. Average values for **f** Ser-2814/RyR2-, **g** Thr-17/PLB-, **i** Ser-2809/RyR2- and **j** Ser-16/PLB-phosphorylation

**Fig. 8:**
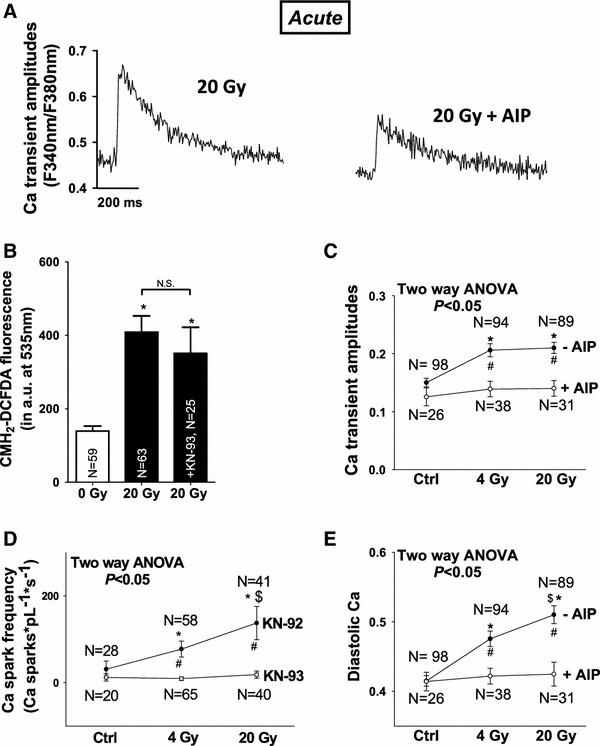
CaMKII-inhibition attenuates irradiation-induced perturbation in Ca handling. **a** Shows original Ca transient traces after irradiation with or without CaMKII-inhibition (AIP) **b** summarizes unaltered ROS-fluorescence following CaMKII-inhibition (KN-93). Average values for **c** Ca transient amplitudes, **d** Ca spark frequency, and **e** diastolic Ca

Data are presented as mean ± SEM. One-way ANOVA combined with Newman–Keuls post hoc tests was performed for Figs. 1, 2, 3, 4, 5, 6b, c, 7f, g, i, j and 8b. Fisher’s exact test was performed to assess significance regarding the fraction of cells that show Ca sparks. Two-way ANOVA combined with Newman–Keuls post hoc tests was performed for Figs. 6e–h,7b, c and 8c–e. Values of *P* < 0.05 were considered as statistically significant.

## Results

### Irradiation regulates cardiac Ca handling

Irradiation of isolated cardiac myocytes induced an acute (~1 h after IR) and dose-dependent positive inotropic response as depicted by increased Ca transient amplitudes obtained from three representative cardiac myocytes after irradiation (Fig. 1a). Mean values for Ca transients (Fig. 1b), fractional myocytes shortening (Fig. 1c) and accelerated Ca transient decay (Fig. 1d) underline the positive inotropic and lusitropic effects of acute irradiation. While left ventricular (LV) ejection fraction and LV internal dimensions were acutely not altered following cardiac irradiation (see Fig. 2c; Table 1), early and highly sensitive measures of alterations in LV contractility [2], i.e., longitudinal as well as radial strain increased immediately (Fig. 2a, b; Table 1). Conversely, when isolating cardiac myocytes from mouse hearts that were irradiated in vivo, we observed a conserved positive inotropic stress response with increased Ca transients and accelerated relaxation (see supplementary Fig. 1a–c). We then examined the duration of the positive inotropic effect on cardiac Ca handling by isolating cardiac myocytes from mice that were irradiated 1 week before cell isolation (i.e., chronic). As shown by the original measurements of Ca handling in Fig. 1e, Ca handling properties had now changed in terms of significantly decreased Ca transients (Fig. 1f), slowed relaxation kinetics (Fig. 1h) and unaltered myocyte contractility (Fig. 1g). Accordingly, echocardiographical measurements of cardiac contractility were found to be not enhanced anymore (see Fig. 2d–f; Table 2) which indicates a biphasical regulation of cardiac Ca handling following cardiac radiation exposure.

**Table 1:** Echocardiographic findings of cardiac function following targeted irradiation in the acute setting

Acute _(after ~20 min)_	0 Gy (*n* = 6)	4 Gy (*n* = 5)	20 Gy (*n* = 6)	OW-ANOVA
Conventional measures				
Heart rate (bpm)	426 ± 9	408 ± 7	442 ± 12	NS
LVESD (in mm)	3.32 ± 0.07	3.29 ± 0.03	3.13 ± 0.08	NS
LVEDD (in mm)	4.50 ± 0.05	4.48 ± 0.05	4.41 ± 0.07	NS
LVESV (in μL)	44.9 ± 2.1	43.8 ± 1.1	39.2 ± 2.4	NS
LVEDV (in μL)	92.7 ± 2.2	91.9 ± 2.6	88.3 ± 3.4	NS
Stroke volume (in μL)	47.8 ± 1.5	48.0 ± 1.6	49.2 ± 2.8	NS
EF (in %)	51.6 ± 1.7	52.3 ± 0.4	55.6 ± 2.3	NS
FS (in %)	26.3 ± 1.1	26.7 ± 0.3	28.9 ± 1.6	NS
Strain measures				
Longitudinal strain	−13.2 ± 0.8	−15.7 ± 0.7	−17.6 ± 1.0	<0.05
Longitudinal strain rate	−4.9 ± 0.3	−5.4 ± 0.4	−6.5 ± 0.7	NS
Radial strain	22.1 ± 2.1	22.5 ± 1.2	32.1 ± 3.0	<0.05
Radial strain rate	6.4 ± 0.5	6.3 ± 0.7	8.8 ± 0.7	<0.05

**Table 2:** Echocardiographic findings of cardiac function following targeted irradiation in the chronic setting

Chronic _(after ~1 week)_	0 Gy (*n* = 5)	4 Gy (*n* = 5)	20 Gy (*n* = 5)	OW-ANOVA
Conventional measures				
Heart rate (bpm)	413 ± 21	422 ± 20	435 ± 12	NS
LVESD (in mm)	3.38 ± 0.11	3.29 ± 0.03	3.30 ± 0.02	NS
LVEDD (in mm)	4.58 ± 0.08	4.48 ± 0.03	4.49 ± 0.05	NS
LVESV (in μL)	47.1 ± 3.9	43.7 ± 0.9	44.1 ± 0.7	NS
LVEDV (in μL)	96.6 ± 3.8	91.6 ± 1.4	92.2 ± 2.5	NS
Stroke Volume (in μL)	49.5 ± 1.3	47.9 ± 1.6	48.1 ± 2.0	NS
EF (in %)	51.5 ± 2.2	52.3 ± 1.2	52.1 ± 0.8	NS
FS (in %)	26.3 ± 1.4	26.7 ± 0.7	25 5 ± 0.5	NS
Strain measures				
Longitudinal strain	−12.4 ± 0.5	−12.4 ± 1.1	−11.5 ± 0.5	NS
Longitudinal strain rate	−4.9 ± 0.3	−5.4 ± 0.4	−6.5 ± 0.7	NS
Radial strain	18.1 ± 2.1	19.6 ± 1.7	16.9 ± 0.6	NS
Radial strain rate	6.0 ± 0.7	5.6 ± 0.6	5.9 ± 0.6	NS

### Irradiation induces persistently disturbed SR Ca handling

Since the amplitude of systolic Ca transients largely depends on SR Ca load, this was investigated in a next step. SR Ca load was acutely not altered by irradiation (Fig. 3a, b). Fractional SR Ca release, however, which describes the relation between the amplitude of a normal Ca transient during contraction and the caffeine-induced Ca transient (SR Ca content) was elevated suggesting increased fractional SR Ca release per systole (Fig. 3c), which most likely underlie the acute increase in Ca transient amplitude in irradiated cells. Acutely activated Ca release from the SR in irradiated cells was also present during diastole in terms of an increased frequency of spontaneous diastolic Ca release events (Ca sparks) that were dose-dependently increased following in vitro (Fig. 3d, e) as well as in vivo irradiation (see suppl. Fig. 1E). Furthermore, the fraction of myocytes that showed Ca sparks was increased in irradiated cells at 4 and 20 Gy (Fig. 3f). This was associated with increasing diastolic Ca levels (Figs. 1a, 4a, suppl. Fig. 1D), and decreased diastolic sarcomere lengths (Fig. 4b). Since SR Ca content was acutely not diminished, increased trans-sarcolemmal Ca influx should have contributed to cytosolic Ca overload. We found [Na]_i_ to be dose-dependently increased following irradiation (Fig. 4c, d) which would favor Na-dependent Ca overloading via the NCX. And indeed, when blocking reverse mode of NCX (using KB-R7943), diastolic Ca was significantly decreased following IR at 4 and 20 Gy (by ~15 and ~11 %, respectively, *P* < 0.05). 24 h after irradiation, cultured cells revealed that diastolic SR Ca leakage persisted (Fig. 5a, b), which was associated with a significantly decreased SR Ca content (Fig. 5c, d). In line with that, we also found diminished SR Ca content in irradiated cells in the chronic setting (Fig. 5e, f).

### Irradiation acutely induces oxidative stress in cardiac myocytes

We next aimed to identify potential pathomechanisms by which IR might have initiated its detrimental effects on cardiac Ca handling. As mentioned above, irradiation involves the induction of cell-toxic ROS in targeted tissues [9]. In line with that concept, we also found a dose-dependently increased ROS-fluorescence in cardiac myocytes following irradiation (Fig. 6a, b). We verified this single cell-based approach by performing ESR-spectroscopy using CMH as probe for the detection of global ROS and found its signal to be dose-dependently elevated as well (Fig. 6c). Pathologically increased oxidative stress is known to alter cardiac Ca handling and in particular to activate SR Ca release [31]. Therefore, we tested in a next step whether ROS-scavengers attenuate IR-dependent effects on Ca handling. We chose to use the generally accepted ROS-scavenger melatonin (Mel) [20]. Mel preincubation significantly attenuated increases in ROS-fluorescence following irradiation (Fig. 6e). As a consequence, decreased SR Ca release became apparent by an attenuated positive inotropy in irradiated myocytes (i.e., diminished systolic Ca transients, Fig. 6d, f) as well as by significantly reduced diastolic SR Ca leakage (Fig. 6g) and attenuated diastolic Ca overloading (Fig. 6h).

### CaMKII activity mediates IR-dependent effects on cardiac Ca handling

Finally, we tested whether increased oxidative stress following IR might have been amplified and maintained by oxidatively activated protein kinases such as Ca/calmodulin-dependent protein kinase II (CaMKII) or cAMP-dependent protein kinase A (PKA) [24]. We found an acutely and dose-dependently activated CaMKII (Fig. 7a) to be persistently increased during the observational period (until 1 week after IR), when also oxidatively activated CaMKII [10] was increased by ~30 % in both groups of irradiation (from 1.00 ± 0.07 at 0 Gy to 1.36 ± 0.10 at 4 Gy, and to 1.28 ± 0.06 a.u. at 20 Gy *P* < 0.05 using OW-ANOVA, see suppl. Fig. 2A, B). This was associated with a hyperphosphorylation of the CaMKII target proteins RyR2 (at the CaMKII-specific phosphorylation site Ser-2,814, Fig. 7e, f) and PLB (at Thr-17, also Fig. 7e, g), whereas PKA-dependent RyR2- or PLB-phosphorylation was unaltered (at Ser-2809 and Ser-16, respectively, see Fig. 7h–j). While NCX-expression was unchanged (suppl. Fig. 2C, D), SERCA2a expression was significantly decreased in the chronic setting (suppl. Fig. 2C, E). This might have contributed to the slowed relaxation kinetics as shown in Fig. 1h. Interestingly, CaMKII-inhibition (using 5 μmol/L AIP, Fig. 7b) as well as Mel prevented acute CaMKII-activation following irradiation, which suggests CaMKII to be a downstream target of acutely elevated ROS in irradiated cells (Fig. 7c). CaMKII-inhibition itself did not affect acutely increased oxidative stress (Fig. 8b). However, CaMKII-inhibition resulted in an attenuation of IR-dependently enhanced SR Ca release in terms of decreased Ca transient amplitudes (Fig. 8a, c) reduced diastolic SR Ca release (Fig. 8d). Moreover, it also attenuated diastolic Ca overloading (Fig. 8e), which suggests activated CaMKII does contribute to the alterations in cardiac Ca handling as induced by IR.

## Discussion

Our study provides evidence that ionizing radiation regulates cardiac myocytes Ca handling. This resulted in an acute positive inotropic stress response that was partly reversed in the long-term (after 1 week), when signs of systolic (e.g., decreased Ca transients) and diastolic dysfunction (e.g., impaired relaxation) arose. We identified an acute and dose-dependent ROS-burst (as induced by IR) to immediately disturb myocytes Ca homeostasis and to activate CaMKII. As a consequence, CaMKII caused site-specific hyperphosphorylation of the SR Ca release channels (at RyR2 Ser-2814), which was associated with persistent SR Ca leakage, decreased SR Ca load and dysfunctional Ca handling.

### Irradiation biphasically regulates cardiac Ca handling

Radiation exposure to tissue represents a supposedly cell-toxic intervention at doses of 4 and 20 Gy that are very well within the range of radiation that is cumulatively received by the heart during chest radiotherapy. In line with the concept that tissue irradiation involves the formation of ROS, we also found oxidative stress to be dose-dependently increased in irradiated cardiac myocytes, which we verified by the use of a single cell fluorescence approach (CM-H_2_DCFDA) as well as by a method that detects global ROS in multicellular preparations (ESR). Cardiac Ca pumps and transporters are subject to redox-dependent modifications that might contribute to dysfunctional Ca handling in cardiac disease states [24]. RyR2 is known ROS-targets that has increased channel open probability upon oxidation [30]. We observed immediately enhanced SR Ca release as a consequence of IR-dependently increased oxidative stress during systole, when increased fractional release resulted in augmented Ca transient amplitudes. This caused a somewhat unexpected initial increase in myocytes sarcomere shortening, whose physiological impact, however, was also apparent in the in vivo setting, where highly sensitive measures of LV contractility (i.e., strain) became immediately increased. We did not directly assess RyR2-oxidation upon IR. Since, ROS-scavenging strongly reduced IR-dependently enhanced SR Ca release, this suggests that redox-modified RyR2 might be indeed centrally involved in the initiation of IR effects on cardiac Ca handling.

Enhanced SR Ca release was also continuously apparent during diastole in terms of an increased frequency of spontaneous Ca sparks, which further underlines the functional relevance of IR-dependently modified RyR2. Ca spark frequency, but not the fraction of sparking cells rose dose-dependently upon irradiation and therefore in parallel with increased oxidative stress, which points to an aggravated leakiness of the RyR2 at high doses of irradiation. Interestingly, while ‘leaking’ Ca from the SR was initially not relevant with respect to the amplitude of systolic Ca transients in the face of a maintained SR Ca content (that was still compensated by an increase in SR Ca reuptake as approximated by accelerated Ca transient decay in irradiated myocytes), it became relevant in the long-term. Already in cultured irradiated myocytes (i.e., after 24 h), persistent SR Ca leakage led to a significant drop in SR Ca content. This was also true in the chronic setting (after 1 week), when decreased Ca transient amplitudes were observed along with typical signs of diastolic dysfunction (i.e., slowed relaxation kinetics), which might in addition prone the irradiated heart to diastolic dysfunction [16]. Interestingly, we found acutely shortened sarcomere lengths most likely due to [Na]_i_-dependent diastolic Ca overload, which might be a result of a ROS-dependently activated ‘late component’ of the sodium current [31]. Taken together, we interpret the overall-picture of IR-dependently disturbed Ca handling as a potentially early sign of myocytes systolic and diastolic dysfunction.

### CaMKII activity contributes to irradiation-induced perturbation of Ca handling

Increased oxidative stress can be sensed, amplified and maintained by ROS-regulated protein kinases, typical examples of which are CaMKII [10] and PKA [5]. Both ‘stress’ kinases could likely have contributed to the observed effects of IR on Ca handling, because they are known to biphasically alter SR Ca handling. They acutely enhance both, SR Ca release and reuptake, which likely happened since SERCA2a is usually negatively regulated by increases in ROS [32]. In contrast, if *chronically* activated, CaMKII- and/or PKA-dependent hyperphosphorylation of RyR2 can cause persistent SR Ca leakage, SR Ca depletion and consecutive cellular dysfunction as typically seen in diseased myocytes [24]. Our western blot data suggest CaMKII as the predominant downstream mediator of IR-dependently increased ROS. CaMKII was acutely activated (Ca-dependent activation) and caused a persistent hyperphosphorylation of the RyR2 (at Ser-2814) and PLB (at Thr-17) until 1 week after IR when also oxidatively activated CaMKII [10] was found to be increased. This most likely caused the acute activation of SR Ca cycling (reuptake and release), whether in the chronic setting also altered levels of protein expression (down-regulated SERCA2a) could have contributed with respect to slowed relaxation kinetics despite Thr-17 hyperphosphorylation. In contrast, PKA-specific phosphorylation sites (RyR2 Ser-2809 and PLB Ser-16) were found to be unaltered. Our western blot data further suggest that an initial ROS-burst is required for the acute Ca-dependent activation of CaMKII, since this was largely prevented in case of melatonin pretreatment (indicating that ROS effects leading to diastolic Ca overloading preceded CaMKII-activation). We also functionally verified the involvement of CaMKII by our pharmacological inhibition experiments in which we showed that CaMKII-inhibition resulted in a large attenuation of acutely enhanced Ca release from the SR (decreased Ca transients and reduced SR Ca leakage). Interestingly, diastolic Ca overload was also decreased following CaMKII-inhibition. This is interesting insofar as activated CaMKII, which is known to contribute to Na-dependent Ca overload upon oxidative stress via an enhancement of late I_Na_, might have played a role [31] in this setting as well.

### Pathophysiological implications limitations and future perspectives

An increasing number of clinical studies highlight the dose-dependent correlation between radiation exposure to the heart as applied during chest radiotherapy and subsequent cardiac morbidity [18]. However, little is known about vascular-independent [12] myocardial pathomechanisms that might underlie RICM, which notably might involve virtually all ROS-sensitive cardiac cell types as well as the contractile machinery of myocytes in its entirety [14].

In that regard, our study is the first to show that IR can persistently hamper cardiac myocytes Ca handling, which involves distinct posttranslational modifications of important Ca handling proteins that can lead to systolic and diastolic myocytes’ dysfunction. We, therefore, suggest that cardiac myocyte Ca handling itself might contribute to RICM independent of effects secondary to vascular injury. Remarkably, the pattern of combined systolic and diastolic dysfunction as seen in myocytes Ca handling in the long-term would resemble some typical clinical features of RICM [6]. Importantly, our study also clearly challenges the contemporary view of cardiac myocytes as being fairly radio-resistant. We identified an initially even potentially compensatory stress-activation of the ROS/CaMKII-dependent pathway [10] to induce persistent SR Ca leakage, diminished SR Ca content and subsequently hampered Ca fluxes in the long-term. Interestingly, that might also represent an arrhythmogenic mechanism [23]. The observed effects of IR on Ca handling might be aggravated in older and already diseased patients, when ROS-homeostasis is already disturbed [15] and CaMKII already activated [27]. Also because of this, CaMKII-inhibition might represent a useful therapeutic instrument, [27] because it would supposedly not affect the anti-cancer actions of elevated ROS, while it might protect the heart from IR-induced Ca mismanagement. Radiation therapy is often combined with chemotherapy and is known to potentiate anthracyclines’ cardiotoxicity [26] that is also associated with disturbed Ca handling and increased oxidative stress [33]. We could recently demonstrate that doxorubicin exposure induces SR Ca leakage via increased ROS and activated CaMKII, which also resulted in dysfunctional Ca handling [22]. Based on this quite striking resemblance, one could speculate whether some of the clinically observed aggravation of radiation on chemotherapy-induced cardiac side effects may result, at least in part, from similar effects of combined radiochemotherapy on cardiac myocytes Ca handling.

In contrast, a considerable limitation of this study could be that modern radiation therapy is usually applied in fractionated and, therefore, lower doses over a longer time span. This might substantially change the pattern of ROS-homeostasis and, therefore, cause a different functional and temporal outcome with respect to Ca handling as compared to the one that we observed in our model.

Nevertheless, our study shows that cardiac Ca handling is subject to ionizing radiation. It may, therefore, help to begin the process of generating and testing hypothesis on the mechanisms of systolic and diastolic dysfunction noted in the patients who have received chest irradiation that may involve changes in cardiocellular ROS-homeostasis and subsequently dysfunctional cardiocellular Ca and Na handling.

## Electronic supplementary material

Below is the link to the electronic supplementary material.

Supplementary material 1 (DOCX 421 kb)
