# Supplementary material for: Ionizing radiation regulates cardiac Ca handling via increased ROS and activated CaMKII
Source: Basic Res Cardiol. 2013 Sep 26;108(6):385. doi: 10.1007/s00395-013-0385-6 (PMC3898380; doi:10.1007/s00395-013-0385-6)
Supplement: Supplementary file 1 — Supplementary material 1 (DOCX 421 kb) [file 395_2013_385_MOESM1_ESM.docx]

**Supplementary Fig. 1: Conserved positive inotropic response in cardiac myocytes isolated from *in vivo* irradiated hearts**

**A:** Original Ca traces obtained from 3 representative cardiac myocytes showing increased Ca transient amplitudes following cardiac irradiation *in vivo*. Average data for **B:** Ca transients, **C:** Ca transient decay, **D:** Diastolic Ca and **E:** Ca spark frequency.

**Supplementary Fig. 2: Oxidatively activated CaMKII, unaltered NCX and down-regulated SERCA2a expression in the long-term following IR.**

**Fig. A** shows original western blot images of increased ox-CaMKII. Averages data for A are shown in **B**. **Fig. C** shows original western blots for NCX-expression and SERCA2a. Average data are shown in **D&E.**
